# Multimodal MRI in Neurodegenerative Disorders

**DOI:** 10.1155/2012/287891

**Published:** 2011-10-16

**Authors:** Antonio Cerasa, Andrea Cherubini, Patrice Peran

**Affiliations:** ^1^Neuroimaging Research Unit, Institute of Neurological Sciences, National Research Council, 88100 Catanzaro, Italy; ^2^Inserm, Imagerie Cérébrale et Handicaps Neurologiques, UMR 825, 31059 Toulouse, France; ^3^Université de Toulouse (UPS), Imagerie Cérébrale et Handicaps Neurologiques, UMR 825, 31059 Toulouse, France

In the last twenty years, advanced magnetic resonance imaging (MRI) techniques have provided fundamental knowledge about neurodegenerative processes underlying several neurological and psychiatric diseases.

The native approach in this field of study was the investigation of a single MRI parameter at a time: (a) blood oxygenation-level-dependent (BOLD) images, (b) anatomical 3D T1-weighted images, (c) diffusion-weighted imaging (DWI)/diffusion tensor imaging (DTI), and (d) quantitative relaxometry. (a) The common observation in functional MRI studies is that increasing in the BOLD MRI signal represents increasing of neural activity. Such a neurophysiological “activation” results from elevated oxygen saturation levels (and reduced paramagnetic deoxyhemoglobin contents) of capillary and venous blood. These positive signal changes are considered the basis for the functional organization of the brain. (b) The T1-weighted sequence allows the employment of several neuroanatomical techniques able to describe and quantify macrostructural changes by using probabilistic (voxel-based morphometry analysis) or quantitative research tools (manual/automatic region-of-interest volumetry, cortical thickness measurements). (c) DWI and DTI provide specific quantitative measurement of micro-structural changes within white and gray matter compartments. In particular DTI provides quantitative parameters, such as the mean diffusivity that increases with microscopic barrier disruption and extracellular fluid accumulation and the fractional anisotropy that provides information on the microstructural integrity of highly oriented microstructures (i.e., myelin). (d) Finally, another potential MRI technique is the quantification of mineral levels in the brain. MR relaxometry is a sensitive method to evaluate the brain iron content *in vivo*. Iron accumulation has been implicated in the pathogenesis of many neurodegenerative diseases.

In the last five years, multimodal neuroimaging has became the most popular approach to study pathophysiology of diseased brain. In fact, the aim of this field of study is to quantify the single or combined weight of MRI parameters in describing neurodegenerative processes. In other words, the possibility to measure MR parameters sensitive to complementary tissue characteristics (e.g., volume atrophy, iron deposition, and microstructural damage) could have great potential for investigating pathological changes in several neurologic/psychiatric diseases. At this moment, important new findings have been reported although several issues remain open. In this special edition, we have featured some papers that address such issues. 

The first paper of this special edition is more forward-looking. In fact, it presents a new experimental multimodal MRI approach to better elucidate interrelated gray and white matter changes in schizophrenic patients. This new method represents a theoretical evolution of the previous and well-known voxel-based analysis of T1-weighted images that generally provides a mixed measure of neuronal “volume” or “density.” 

The second and third papers provide fundamental overviews of recent findings about neurodegenerative processes underlying Alzheimer's disease (AD) and mild cognitive impairments (MCIs) obtained through a multimodal neuroimaging approach. The subsequent paper addresses the specific advance in functional neuroimaging studies of AD and MCI patients. All these papers highlight the importance of multimodal neuroimaging in enhancing our ability to diagnose MCI and AD in its early stages. 

Finally, the last two papers present the application of advanced MRI measurements (automatic ROI volumetry, cortical thickness measurements, and T2* iron quantification) to define the presence of new biomarkers able to distinguish AD patients from normal pressure hydrocephalus and to improve the accurate diagnosis of suspect neuroferritinopathy, a neurodegenerative disorder characterized by the deposition of iron and ferritin in the brain.

*Antonio Cerasa* *Antonio Cerasa* *Andrea Cherubini* *Andrea Cherubini* *Patrice Peran* *Patrice Peran*

